# Risk factors for hypertension among young adults (18-35) years attending in Tenwek Mission Hospital, Bomet County, Kenya in 2018

**DOI:** 10.11604/pamj.2019.33.210.18407

**Published:** 2019-07-16

**Authors:** Damaris Ogake Ondimu, Gideon Mutie Kikuvi, Walter None Otieno

**Affiliations:** 1 Institute of Tropical Medicine, Jomo Kenyatta University of Agriculture and Technology, Nairobi, Kenya; 2 Maseno University, School of Medicine, Maseno, Kenya

**Keywords:** Risk factors, hypertension, young adults, cases

## Abstract

**Introduction:**

Hypertension ranks third in the world, after underweight and unsafe sex, in the list of six major risk factors contributing to the global disease. In Kenya, the prevalence stands at 24% in the general population, while among the young adults, the incidence of hypertension has been reported to be in the rise; a fact attributed to increased number of risks. We therefore sought to determine awareness and risk factors of hypertension among young adults attending Tenwek hospital.

**Methods:**

A case-control study of young adults ages 18-35, involving 80 cases and 80 controls at Tenwek Mission Hospital, Bomet County. Cases included males and females newly diagnosed with hypertension (diagnosed at the time of data collection) and if they reported taking antihypertensive medication and reported as hypertensives in the hospital records at any clinic visit or at interview, while controls included persons with no history of hypertension.

**Results:**

Those having a BMI≥25 were 3.05 times more likely to be hypertensive (OR: 3.05, 95% CI 1.26, 7.40; p=0.014). Having a relative suffering from hypertension increased almost thrice the odds of being hypertensive (OR: 2.78, 95% CI 1.20, 6. 46; p=0.018). Not drinking alcohol reduced the chance of suffering from hypertension by 70%, (OR=0.30, 95% CI 0.11, 0.81; p=0.017).

**Conclusion:**

The prevalence of hypertension in younger adults is not as low as generally perceived. Preventive measures should be formulated in a manner to address variety of major risk factors in young adults.

## Introduction

Hypertension, also called high blood pressure, is a condition that arises when the body's smaller blood vessels (the arterioles) narrow, causing the blood to exert excessive pressure against the vessel walls and forcing the heart to work harder to maintain the pressure [1]. Usually hypertension is described as a systolic blood pressure (SBP) of 140 mm Hg or more, or a diastolic blood pressure (DBP) of 90 mm Hg or more [2]. Blood pressure for adults aged 18 years or older can be classified as follows; normal: systolic lower than 120 mm Hg, diastolic lower than 80 mm Hg, prehypertension: systolic 120-139 mm Hg, diastolic 80-89 mm Hg, stage 1: systolic 140-159 mm Hg, diastolic 90-99 mm Hg, stage 2: systolic 160 mm Hg or greater, diastolic 100 mm Hg or greater [3]. Various physiological changes occur including atherosclerosis and vascular changes that occur with aging are responsible for most occurrence of hypertension in the older populations. Therefore advanced age has been associated with increasing incidences of hypertension [4-6]. Global prevalence of hypertension is 31%. It has been estimated that premature deaths resulting from hypertension annually are approximately 7.1 million, which account for 64 million Disability-Adjusted Life Years (DALYs) [7]. Hypertension ranked third in the world, after underweight and unsafe sex, in the list of six major risk factors contributing to the global disease burden [8]. In Kenya the prevalence stands at 24% of the population [7]. Young adult comprises of 35% of Kenyan population (Kenya National Bureau Of statistics (KNBS) 2009 census). National Institute of Health (NIH) in a global multicentre study found out that there are 19% young adults with hypertension than previously believed. The new study which took blood pressure readings of 14,000 men and women aged between 24 and 32 years revealed a higher percentage of high blood pressure readings than results from previous studies [9]. Hypertension has become a public health concern in sub-Saharan Africa; Kenya included [10-12]. It is also a major non-infective factor in the high mortality of adults in sub-Saharan Africa [13]. The risk factors for hypertension are increasing in rural and urban populations [14]. These risk factors include smoking, alcohol intake and sedentary lifestyles. Further, cross-sectional studies have only demonstrated weak health care systems as the major and sole factor responsible for the observed rising incidence of hypertension within the young population in rural settings in Africa [15]. Therefore, this study sought to find the risk factors of hypertension among young adults within the catchment of Tenwek Mission Hospital.

## Methods

This was case control study in which young adults, ages between 18-35 years attending in Tenwek Mission Hospital were eligible for the study. Tenwek is in Bomet County, lies between latitudes 0º 29' and 1º 03' south and between longitudes 35º 05' and 35º 35' east. It is Christian mission hospital offering primary healthcare to a population of about 800,000 and a surgery center for a region of more than 8.5 million people. The researcher and research assistant visited the health facility every day during the one month period of the survey to meet the sample size recommended in the study. Patients walked into the clinic, their clinical records were examined to assess their eligibility.

**Case definition:** individuals were classified as cases at the time of visit if they had a systolic blood pressure ≥140 mm Hg or a diastolic blood pressure ≥90 mm Hg on at least two clinic visits or if they reported taking antihypertensive medication at any clinic visit or at interview. Controls were defined as other patients attending the same outpatient service with no prior history of hypertension and the blood pressure was normal on the date of interview.

**Sample size determination:** with the proportion of those exposed in the control group being 24% from the literature [7]. We sought to know the minimum sample size sufficient to detect an odds-ratio of 2.7 or greater. We assumed 80% statistical power, with equal number of cases and controls (r=1). The Fleiss formula (Fleiss, 1981) for calculating sample size for case control studies was used.

$$n=\left(\frac{r+1}{r}\right)\frac{\left(\bar{p}\right)\left(1-\bar{p}\right){\left(z_{\beta }+{\text{Z}}_{\alpha /2}\right)}^{2}}{{\left({\text{p}}_{1}-p_{2}\right)}^{2}}$$

For 80% power, Z_β_=.84 For 0.05 significance level, Z_α_=1.96 r=1 (equal number of cases and controls). The proportion exposed in the control group is 24%. To get proportion of cases exposed n=2*(0.32)(1-0.32)*(0.84+1.96)^2/(0.45-0.24)^2 =3.412/0.441=70.49. When we factor in 10% refusal or spoilt questionnaires, the minimum sample size required = 78 for each group. We therefore recruited a total of 160 respondents both male and female aged between 18-35 consisting of 80 cases and 80 controls were studied.

**Data management and analysis:** the questionnaires were administered by the interviewer after explaining the purpose of study and obtaining an informed consent. Completed questionnaires were received, checked for completeness and entered using Access forms. Data cleaning and analysis was done using STATA version 14.1 (STATA Corporation, College Station, Texas, USA). The overall socio-demographic characteristic of the study population was analyzed using frequencies and proportions. Prior to analysis, some variables were recoded, collapsed and others combined. For example, highest education level attained was re-recoded combining tertiary with university because of the low number in those categories. Those who refused to answer a specific question “refused to answer” or answered, “Don't know” were set to missing where necessary in order to exclude them from the analysis. Those who answered “Don't know” for the question “Are you currently on medication?” were set to missing in order to exclude them from the analysis. Pearson's Chi-square or Fisher's exact test were used to test for the association between the hypertension and categorical variables. Unconditional logistic regression was fitted to find out risk factors of hypertension. We fitted both the unadjusted and adjusted model. All covariates with p-value ≤ 0.1 or set a prior were included in the adjusted model. All estimates were done at 5% level of significance.

## Results

**Socio-demographic characteristics of the study participants:** the highest percentage of respondents were in the age group of 30-35 years which comprised of cases and controls (60% vs. 40.3% respectively; p < 0.041). Seventy one percent of the respondents resided in the rural area. Six two percent (62%) respondents reported having acquired secondary and tertiary level of education. Majority of respondents (71.5%) were married. Majority (53%) reported earning some income and 42% of respondents earned an average monthly income of none or less than Ksh 10,000 (Table 1).

**Table 1:** Socio-demographic characteristic of the study population (N=152)

		Cases	Controls	
	Total			P
Factors/Variables	n (%)	n (%)	n (%)	
**Age group(years)**				
18-23	26 (17.7)	9 (12.0)	17 (23.6)	
24-29	47 (32.0)	21 (28.0)	26 (36.1)	
30-35	74 (50.3)	45 (60.0)	29 (40.3)	0.041
**Highest level of education attained**				
Primary or less	31(20.5)	14 (18.4)	17 (22.7)	
Secondary	26 (17.2)	16 (21.1)	10 (13.3)	
Tertiary/University	94 (62.3)	46 (60.5)	48 (64.0)	0.425
**Marital status**				
Single	43 (28.5)	20 (26.7)	23 (30.3)	
Married	108 (71.5)	55 (73.3)	53 (69.7)	0.624
**Occupation**				
None/Student	19 (12.6)	8 (10.7)	11 (14.5)	
Farmer	36 (23.8)	18 (24.0)	18 (23.7)	
Earn Income	80 (53.0)	39 (52.0)	41 (54.0)	
Unskilled	16 (10.6)	10 (13.3)	6 (7.9)	0.678
**Average monthly income**				
non/<10000	60 (42.6)	31 (46.3)	29 (39.2)	
11000-30000	41 (29.0)	20 (29.9)	21 (28.4)	
31000-60000	40 (28.4)	16 (23.8	24 (32.4)	0.510
**Area of residence**				
Urban	21 (14.1)	12 (16.0)	9 (12.2)	
Rural	105 (70.5)	52 (69.3)	53 (71.6)	
Peri-urban	23 (15.4)	11 (14.7)	12 (16.2)	0.789
**Body mass index (BMI)**				
<=24.9	60 (47.6)	22 (34.9)	38 (60.3)	
>25	66 (52.4)	41 (65.1)	25 (39.7)	0.004
**Smoking**				
No	27(23.7)	10 (18.2)	17 (28.8)	
Yes	87 (76.3)	45 (81.8)	42 (71.2)	0.182
**Alcohol**				
No	34 (27.4)	12 (18.8)	22 (36.7)	
Yes	90 (72.6)	52 (81.3)	38 (63.3)	0.025
**Obesity**				
No	32 (25.4)	14 (23.3)	18 (27.3)	
Yes	94 (74.6)	46 (76.7)	48 (72.7)	0.612

### Inferential data analysis

**Known risk factors of hypertension:** among the known risk factors (alcohol, obesity, smoking and family history), only obesity and family history were statistically significant in this study (Table 2).

**Table 2:** The distribution of the known hypertension risk factors across cases and controls

		Control	Case	
Variable/Factor	Total (N)	n (%)	n (%)	P
**Smoking**				0.113
No	27(18.6)	17(63.0)	10(37.0)	
Yes	87(60.0)	42(48.3)	45(51.7)	
Don't know	31(21.4)	11(35.5)	20(64.5)	
**Alcohol**				0.082
No	34(23.4)	22(64.7)	12(35.3)	
Yes	90(62.1)	38(42.2)	52(57.8)	
Don't know	21(14.5)	10(47.6)	11(52.4)	
**Body mass index**				0.004
<=24.9	60(47.6)	38(63.3)	22(36.7)	
>25	66(52.4)	25(37.9)	41(62.1)	
**Do you have relative is suffering from hypertension**				0.003
No	65(48.1)	41(63.1)	24(36.9)	
Yes	70(51.9)	26(37.1)	44(62.9)	

**Lifestyle risk factors of hypertension:** Table 3 shows the bivariate analysis of lifestyle risk factors of hypertension. Frequency of eating red meat and alcohol consumption increased the risk of developing hypertension. The risk of hypertension tends to increase with frequency of eating red meat. Those who reported not eating red meat at all had 77% less chance of being hypertensive compared to those reporting 1 to 2 times a week (OR=0.23, 95% CI 0.07, 0.70; p=0.010), whereas those respondents reporting eating red meat once were 71% less likely to be hypertensive (OR=0.29, 95% CI 0.12, 0.69; p=0.010) Table 4. In the multivariable analysis, participants who reported not eating red meat at all had 83% less chance of being hypertensive compared to those reporting 1 to 2 times a week (OR=0.17, 95% CI 0.04, 0.64; p=0.012), whereas those respondents reporting eating red meat once per week had 72% less chance to be hypertensive (OR=0.28, 95% CI 0.10, 0.74; p=0.011). Those having a BMI ≥ 25 were 3.05 times more likely to be hypertensive (OR: 3.05, 95% CI 1.26, 7.40; p=0.014). Having a relative suffering from hypertension increased almost thrice the odds of being hypertensive (OR: 2.78, 95% CI 1.20, 6. 46; p=0.018). Not drinking alcohol reduced the chance of suffering from hypertension by 70%, (OR=0.30, 95% CI 0.11, 0.81; p=0.017).

**Table 3:** Bivariate analysis of lifestyle risk factors of hypertension

	Cases	Controls		
Factors/Variables	n (%)	n (%)	OR (95% CI)	p
**How often do eat red meat**				0.010
Never	15 (20.0)	6 (8.2)	0.23 (0.07, 0.70)	
<1/wk	20 (26.7)	34 (46.6)	0.29 (0.12, 0.69)	
1-2/wk	26 (34.7)	13 (17.8)	Ref	
>2/wk	14 (18.7)	20 (27.4)	0.84 (0.34, 2.02)	
**How often do you eat processed meat**				0.303
Never	42 (58.3)	35 (50.7)	Ref	
<1/wk	26 (36.1)	25 (36.2)	1.15 (0.56, 2.34)	
>/wk	4(5.6)	9 (13.0)	2.70 (0.76, 9.52)	
**Do you smoke**	42 (71.2)	45 (81.8)	1.82 (0.75, 4.42)	0.317
**Do take alcohol**	38 (63.3)	52 (81.3)	2.51 (1.11, 5.69)	0.028
**How often do you exercise?**				0.139
Always	18 (27.3)	9 (14.1)		
Rarely	38 (57.6)	40 (62.5)	2.11 (0.84, 5.25)	
Never	10 (15.2)	15 (23.4)	3.00 (0.96, 9.30)	
**How often do you add salt in food?**				0.145
Every meal	11 (14.5)	11 (15.1)		
Once in a while	53 (69.7)	49 (67.1)	0.92 (0.36, 2.32)	
I don’t add	12 (15.8)	13 (17.8)	1.08 (0.34, 3.41)	
**Consumption of vegetables**				0.989
Everyday	32 (42.1)	32 (43.2)		
Once in a while	43 (56.6)	41 (55.4)	0.95 (0.49, 1.82)	
I don’t take	1 (1.3)	1 (1.4)	(0.05, 16.6)	

**Table 4:** Multivariate analysis of socio-demographics and socio-economic risk factors of hypertension

	Unadjusted Odds Ratio			Adjusted Odds Ratio		
Factor/Variable	OR	95% CI	p	OR	95% CI	p
**Age group(years)**						
18-23	Ref					
24-29	1.53	(0.57,4.11)	0.404	0.91	(0.28,2.95)	0.878
30-35	2.93	(1.15,7.45)	0.024	2.01	(0.68,5.94)	0.208
**Body mass index (BMI)**						
<=24.9	Ref					
>25	2.83	(1.37,5.84)	0.005	3.05	(1.26,7.40)	0.014
**How often do you eat red meat**						
Never	0.24	(0.08,0.70)	0.01	0.17	(0.04,0.68)	0.012
1/wk	0.29	(0.12,0.70)	0.006	0.28	(0.10,0.74)	0.011
1-2/wk	Ref			Ref		
>2/wk	0.84	(0.35,2.02)	0.698	0.83	(0.30,2.33)	0.727
**How often do you do exercise**						
Always	Ref					
Rarely	2.11	(0.84,5.26)	0.111	2.02	(0.65,6.27)	0.222
Never	3.00	(0.97,9.30)	0.057	3.67	(0.92,14.66)	0.065
**Do you have any relative suffering from Hypertension**						
No	Ref					
Yes	2.89	(1.44,5.82)	0.003	2.78	(1.20,6.46)	0.018
**Do you take alcohol**						
No	0.40	(0.18,0.90)	0.028	0.30	(0.11,0.81)	0.017

## Discussion

Although age did not remain significant in the final multivariable analysis, it emerged as stronger demographic risk factor for hypertension in the bivariate; especially those between the age group of 30-35 years of age. The study findings suggest that hypertension is more prevalent in the rural areas (69.3%) as opposed to urban area (14%). This finding contrasts with numerous studies showing a higher prevalence of hypertension in urban African [16]. More than a half of cases reported earning average monthly income of none or less than Ksh 10,000. There was higher number of hypertension(cases) reported in this income brackets (43.6%) as compared to people with higher income (23.8%) which was not statistically significant (p=0.51). Numerous indices of Social Economic Status (SES) have been studied, stating that SES can be highly variable over time, particularly with regard to income, and it appears that blood pressure level may be sensitive to these fluctuations [15, 17]. Being obese (BMI≥25), frequently eating red meat, having any relative who suffer from hypertension and drinking of alcohol are associated risk factors of hypertension [18]. Non-alcohol drinkers had nearly 70% less chance of suffering from hypertension, compared to those who drank alcohol. There was also a direct relationship between current smoking and hypertension. Smokers were 1.82, (CI 0.75-4.42) higher chance of being hypertensive and wasn't statistically significant. This study conforms with a study of social habits such as smoking, alcohol consumption and physical inactivity, higher anthropometric parameters (BMI and WHR) and diet rich in red meat and low in fruit was associated with hypertension among Kenya defense forces [16, 19].

## Conclusion

The study has shown that hypertension among the young adult is not as low as earlier perceived. Obesity and unhealthy lifestyle behaviors e.g. alcohol use and high meat consumption emerged as the main risk factors in the study population. This appeals for more focused studies for this age group that involves reappraisal on the major aspects of hypertension in young adults, promoting interest and discussions among relevant authorities.

### What is known about this topic

- Most studies have reported age as a stronger demographic risk factor for hypertension, with a direct correlation between advanced age and the risk of developing hypertension;
- However, there is paucity of data regarding actual prevalence of hypertension in young adults.

### What this study adds

- This study showed that prevalence of hypertension in younger adults is not as low as generally perceived. These findings appeals for reappraisal of risk factors of hypertension among young adults.

## Competing interests

The authors declare no competing interests.
